# Analysis of the stability of a predator-prey model including the memory effect, double Allee effect and Holling type-I functional response

**DOI:** 10.1371/journal.pone.0305179

**Published:** 2025-01-03

**Authors:** Ramesh K., Ranjith Kumar G., Aziz Khan, Thabet Abdeljawad

**Affiliations:** 1 Department of Mathematics, Anurag University, Venkatapur, Hyderabad, Telangana, India; 2 Department of Mathematics and General Sciences, Prince Sultan University, Riyadh, Saudi Arabia; 3 Department of Medical Research, China Medical University, Taichung, Taiwan; 4 Center for Applied Mathematics and Bioinformatics (CAMB), Gulf University for Science and Technology, Hawally, Kuwait; 5 Department of Mathematics and Applied Mathematics, Sefako Makgatho Health Sciences University, Garankuwa, Medusa, South Africa; Italian Institute of Technology, ITALY

## Abstract

This study proposes and analyses a revised predator-prey model that accounts for a twofold Allee impact on the rate of prey population expansion. Employing the Caputo fractional-order derivative, we account for memory impact on the suggested model. We proceed to examine the significant mathematical aspects of the suggested model, including the uniqueness, non-negativity, boundedness, and existence of solutions to the noninteger order system. Additionally, all potential equilibrium points for the strong and weak Allee effect are examined under Matignon’s condition, along with the current state of conditions and local stability analysis. Analytical results are also provided for the necessary circumstances for the Hopf bifurcation initiated by the fractional derivative order to occur. We also demonstrated the global asymptotic stability for the positive equilibrium point in both the strong and weak Allee effect cases by selecting an appropriate Lyapunov function. This study’s innovation is its comparative investigation of the stability of the strong and weak Allee effects. To conclude, numerical simulations validate the theoretical findings and provide a means to investigate the system’s more dynamical behaviours.

## 1. Introduction

Investigating the complex nature of the predator-prey framework could supply valuable insights into numerous processes which take place during interactions between predators and their prey. The Allee influence is a significant ecological phenomenon that affects the per capita expansion of predator or prey populations of predator or prey populations. It is associated with the situation in which at low levels of population, the population’s per-capita increase pace is positively correlated with its density. As far as Allee effects are concerned, there are two varieties: strong and weak. The strong Allee impact results in the population to decline below a particular point, known as the Allee level, at the moment when the per capita expansion pace starts to decline [1,2]. More specifically, in low-density populations, the conservation biology-related risk of extinction increases as the Allee threshold becomes bigger. Conversely, in situations when there are few individuals in a population, the rate of increase per individual always remains positive in the weak Allee effect [3–5]. When multiple processes that cause the Allee influence have an impact on the same population at the same time, it’s called the twofold (or multiple) Allee phenomenon [6,7].

For a given population, the most prevalent continuous increase function is given by the following equation when considering a single mechanism and the multiplicative Allee effect:

$$\frac{dx_{1}}{dt}=rx_{1}(1-\frac{x_{1}}{\rho })\left(x_{1}-\alpha \right),$$ (1)

with *x*_1_ standing for population density, *ρ* for carrying capacity and *r* for the population’s inherent per capita expansion rate. An adjustment to the logistic model, the term (*x*_1_−*α*) shows that when *x*_1_<*α*, the population density drops to zero and $\frac{dx_{1}}{dt}<0$, and when *x*_1_>*α*, the population expands to *ρ*, with $\frac{dx_{1}}{dt}>0$. If a certain population level (*α*) is greater than zero or if −*ρ*<*α*≤0, then the impact of Allee in Eq (1) may be specified as strong or weak. Predator-prey interaction models may include multiple processes of Allee influences within the same population, ensuing in a demographic Allee influence [7]. The collective influence of these factors is referred to as a double or multiple Allee effect. The prevalence of two or more component Allee effects was shown by a recent assessment by Berec et al. [7] of the findings for these effects, which included instances from both terrestrial and aquatic ecosystems, as well as from plants, invertebrates, and vertebrates, also well as from both naturally occurring and artificially manipulated populations. The literature on prey-predator interaction models that include the double Allee effect is few, with just a handful of publications covering the topic over the last several decades [8,9]. If the multiplicative double Allee effect were to impact a single population’s growth function, the following equation would control one of its mathematical forms

$$\frac{dx_{1}}{dt}=\frac{rx_{1}}{x_{1}+\beta }(1-\frac{x_{1}}{\rho })\left(x_{1}-\alpha \right),$$ (2)

for any auxiliary parameters *β*>0 resulting in *α*>−*β*. Both the factor) (*x*_1_−*α*) representing the Allee effect, is discussed above for Eq (1), and the hyperbolic function $\frac{rx_{1}}{x_{1}+\beta }$ representing the other Allee effect, which influences the species’ inherent rate of increase due to external challenges (with the supplementary parameter *β*>0 quantifies the intensity of the Allee effect), are present in Eq (2) and both have an impact on the same population growth. The primary goal of the aforementioned research initiatives is to explore the influence of the Allee factor on the occurrence of distinct behaviours in the predator-prey system. Despite evidence suggesting the twofold Allee effect may be seen in predator population increase, the majority of research have only examined its impact on prey population growth [10].

It is impossible for prey and predator interactions to take place in nature without memory, because the paces of prey and predator growth for a specific moment are dependent on the history of the variables at all past moments, rather than just the local state at that moment [11–15]. Determining the fractional-order derivative perfectly captures all of the above requirements. It may represent the memory and genetic features inherent in different processes, which is an advantage over the integer order derivative. Recent years have seen fractional derivatives in fractional calculus emerge as a powerful tool for characterising the memory and genetic features of a wide range of materials and processes [16], in fields as diverse as biology, economics, engineering, physics, and many more [17–21]. A number of researchers have shown that memory-based systems are superior at representing and explaining real-world occurrences [22–28].

In light of all of this, the purpose of this investigation is to analyse the fractional-order predator-prey system that takes the double Allee phenomena into account. To account for the influence of memory on prey and predator growth rates, the suggested framework includes the Caputo fractional-order derivative. It is our understanding that no one else has suggested or explored the complexities of the model we have provided, which includes the memory impact using the Caputo non-integer order derivative and the double Allee influence in the prey increase. It is possible that this study may shed light on hitherto unknown aspects of the multiple Allee effect and point the way for further investigation in a wide range of research methods, including theoretical modelling, field investigations, and laboratory trials.

Here is how the rest of this paper is structured. The system construction, which covers the existence, boundedness, non-negativity, and uniqueness of our system’s solutions, is stated in Section 2. Next, we examine the model’s dynamic behaviours for both weak and strong Allee effects at every possible equilibrium point. The Hopf bifurcation analysis is then provided in section 3. Furthermore, the adequate requirements for the inner equilibrium points’ global stability are met. Section 4 displays numerical simulations that both validate our analytical results and numerically investigate the effects of the fractional-order system’s order, Allee threshold on both strong and weak of our model’s dynamics. We wrap up our findings under Section 5.

## 2. Model formulation

This research takes a look at a prey-predator scenario in which the predator feeds on prey based on its functional response and the prey population grows in response to a twofold Alleeeffect of type (2). Following that, the predator-prey paradigm transforms into

$$\begin{array}{l} \frac{dx_{1}}{dt}=\frac{rx_{1}}{x_{1}+\beta }(1-\frac{x_{1}}{\rho })(x_{1}-\alpha )-ax_{1}x_{2},\\ \frac{dx_{2}}{dt}=bx_{1}x_{2}-cx_{2}, \end{array}$$ (3)

such that *x*_1_>0, *x*_2_>0. In system (3) *x*_1_(*t*) and *x*_2_(*t*) represents the prey and predator densities at time *t*. Table 1 displays the descriptions and symbols of the aforementioned parameters. To capture the whole time condition of population expansion, we consider non-integer derivative order on the left side of the traditional derivative model (3), as shown below:

$$\begin{array}{l} {\;}^{C}D_{t}^{\zeta }x_{1}(t)=\frac{rx_{1}}{x_{1}+\beta }\left(1-\frac{x_{1}}{\rho }\right)(x_{1}-\alpha )-ax_{1}x_{2},\\ {\;}^{C}D_{t}^{\zeta }x_{2}(t)=bx_{1}x_{2}-cx_{2}. \end{array}$$ (4)

**Table 1:** The system’s (3) parameters description.

Parameter	Details of the parameters
*r*	Inherent growth rate of the prey
*ρ*	The prey’s sustainability carrying capacity
*a*	Capturing rate of the predator
*α*	The prey’s longevity threshold
*β*	The auxiliary parameter
*b*	Rate of conversion of prey to biomass of the predator
*c*	The predator’s natural mortality rate

The symbol ${\;}^{C}D_{t}^{\zeta }$ symbolizes the Caputo fractional-order derivative of a real valued function *f* which is defined as follows:

$${\;}^{C}D_{t}^{\zeta }f(t)=\frac{1}{\Gamma (n-\zeta )}\int_{0}^{t}\frac{f^{n}(\vartheta )}{{\left(t-\vartheta \right)}^{n-\zeta -1}}d\vartheta ,$$

where Γ(.) is the Gamma function and *ζ*∈(*n*−1,*n*],*n*∈ℕ [16]. In system (4), all of the parameters are identical to those in system (3).

### 2.1 Existence, uniqueness, non-negativity, and boundedness

**Theorem 1**. *For each $Z_{0}=(x_{1_{0}},x_{2_{0}})\in \Omega$, then the initial value problem of system (4) possesses unique solution Z(t)*∈Ω *that is specified for all t*≥0.

**Proof.** We need to consider the possibility whether the dynamical system having a unique solution in the region [0,∞)×Ω, where Ω = {(*x*_1_,*x*_2_)∈R^2^:max{|*x*_1_|,|*x*_2_|}≤*Q*} if we want to verify this claim. Suppose that

$Z=(x_{1},x_{2}),\bar{Z}=({\bar{x}}_{1},{\bar{x}}_{2})$ and now consider a mapping *V*(*Z*) = (*V*_1_(*Z*),*V*_2_(*Z*)), where $V_{1}(Z)=rx_{1}(1-\frac{x_{1}}{\rho })(\frac{x_{1}-\alpha }{x_{1}+\beta })-ax_{1}x_{2}$ and *H*_2_(*V*) = *bx*_1_*x*_2_−*cx*_2_.Then, for any $Z,\bar{Z}\in \Omega$ we have

$$\left\|V(Z)-V(\bar{Z})\|=|V_{1}(Z)-V_{1}(\bar{Z})|+|V_{2}(Z)-V_{2}(\bar{Z})\right|$$

$$\begin{array}{l} =\left|rx_{1}(1-\frac{x_{1}}{\rho })(\frac{x_{1}-\alpha }{x_{1}+\beta })-ax_{1}x_{2}-r{\bar{x}}_{1}(1-\frac{{\bar{x}}_{1}}{\rho })(\frac{{\bar{x}}_{1}-\alpha }{{\bar{x}}_{1}+\beta })+a{\bar{x}}_{1}{\bar{x}}_{2}\right|\\ +|bx_{1}x_{2}-cx_{2}-b{\bar{x}}_{1}{\bar{x}}_{2}+c{\bar{x}}_{2}| \end{array}$$

$$\begin{array}{l} \leq \left\{r(1-\frac{\left(1+\alpha /\beta \right)}{{\left(1+Q/\beta \right)}^{2}})+\frac{r}{\rho {\left(Q+\beta \right)}^{2}}(2Q^{3}-(\alpha -3\beta )Q^{2}-2\alpha \beta Q)+(a+b)Q\right\}|x_{1}-{\bar{x}}_{1}|\\ +((a+b)Q+c)|x_{2}-{\bar{x}}_{2}|, \end{array}$$

$$\leq L_{1}|x_{1}-{\bar{x}}_{1}|+L_{2}|x_{2}-{\bar{x}}_{2}|,$$

$$\leq L\|V-\bar{V}\|,$$

where *L* = max{*L*_1_,*L*_2_} and

$$\begin{array}{l} L_{1}=r(1-\frac{\left(1+\alpha /\beta \right)}{{\left(1+Q/\beta \right)}^{2}})+\frac{r}{\rho {\left(Q+\beta \right)}^{2}}(2Q^{3}-(\alpha -3\beta )Q^{2}-2\alpha \beta Q)+(a+b)Q,\\ L_{2}=(a+b)Q+c. \end{array}$$

As a result, the mapping *V*(*Z*) fulfils the Lipschitz requirement. Hence, with the given starting condition $Z_{0}=(x_{1_{0}},x_{2_{0}})$, where $x_{1_{0}}\geq 0$ and $x_{2_{0}}\geq 0$ there is a unique solution to the system of differential Eq (4), as determined by the existence and uniqueness theorem of [29].

**Theorem 2.** All solutions to the differential Eq (4) that originate in the region $R_{+}^{2}$ are uniformly bounded and non-negative.

**Proof.** To begin, we look at the region $R_{+}^{2}$ to see whether the solution is non-negative. Consequently, for any *t*≥0, we have to establish that *x*_1_(*t*)≥0 and *x*_2_(*t*)≥0. Let’s say that for any *t*≥0, the condition *x*_1_(*t*)≥0 is false. Then, there exist *t*_1_>0 such that *x*_1_(*t*)>0 for *t*∈[0,*t*_1_),*x*_1_(*t*_1_) = 0 and *x*_1_(*t*^+^_1_)<0.Then, using the first equation in (4), we have $D_{t}^{\xi }x_{1}(t){|}_{t=t_{1}}=0.$ Lemma 1 of [30] suggests that *x*_1_(*t*^+^_1_) = 0, which defies the assertion that *x*_1_(*t*^+^_1_)<0. As a result, we may conclude that *x*_1_(*t*)≥0 for any *t*≥0. Using the same approach, it becomes clear that there is a non-negative solution to Eq (4) with a beginning point in area $R_{+}^{2}$. Consequently, for any *t*≥0, it provides *x*_2_(*t*)≥0 in the same way. After that, we need to prove that the solutions to Eq (4), which begin in the area $R_{+}^{2}$, are uniformly bounded.

**Theorem 3.** *All the solutions to the system begin with $R_{+}^{2}$, are uniformly bounded and enter the region* $\Theta =\{(x_{1},x_{2})\in R_{+}^{2}:0<x_{1}+\frac{a}{b}x_{2}<\frac{H}{\mu }\}$.

**Proof.** For given initial conditions *x*_1_(0)>0,*x*_2_(0)>0, let (*x*_1_(*t*),*x*_2_(*t*)) be the solution of the framework (4) at any time *t*>0.

Consider the function $\omega (t)=x_{1}+\frac{a}{b}x_{2}$ and the non-integer time derivative of this is ${\;}^{C}D_{t}^{\zeta }\omega (t)={\;}^{C}D_{t}^{\zeta }x_{1}+\frac{a}{b}{\;}^{C}D_{t}^{\zeta }x_{2}$,

$$\frac{d^{\zeta }}{dt^{\zeta }}\omega (t)=rx_{1}(1-\frac{x_{1}}{\rho })\frac{\left(x_{1}-\alpha \right)}{x_{1}+\beta }-\frac{acx_{2}}{b}.$$

Now, for *μ*>0 we have

$$\begin{array}{l} \frac{d^{\zeta }}{dt^{\zeta }}\omega (t)+\mu \omega (t)=\frac{-rx_{1}^{3}}{x_{1}+\beta }+\frac{rx_{1}^{2}(1+\alpha )}{x_{1}+\beta }-\frac{r\alpha x_{1}}{x_{1}+\beta }-\frac{ac}{b}x_{2}+\mu x_{1}+\frac{\mu a}{b}x_{2}\\ \;\leq r\left[\frac{-x_{1}^{3}}{x_{1}+\beta }+\frac{x_{1}^{2}(1+\alpha )}{x_{1}+\beta }+(\frac{\mu }{r}-\frac{\alpha }{x_{1}+\beta })x_{1}\right],\\ \frac{d^{\zeta }}{dt^{\zeta }}\omega (t)+\mu \omega (t)<H, \end{array}$$

where $H=\underset{x\in R^{+}}{\max}\{r[\frac{-x_{1}^{3}}{x_{1}+\beta }+\frac{x_{1}^{2}(1+\alpha )}{x_{1}+\beta }+(\frac{\mu }{r}-\frac{\alpha }{x_{1}+\beta })x_{1}]\}>0$ Following Lemma 1 of [31,32], we get

$\omega (x_{1}(t),x_{2}(t))<\frac{H}{\mu }+E_{\zeta }(-\mu t^{\zeta })\omega (x_{1}(0),x_{2}(0))\to \frac{H}{\mu }$ as *t*→∞. As a result, the region $\Theta =\{(x_{1},x_{2})\in R_{+}^{2}:0<x_{1}+\frac{a}{b}x_{2}<\frac{H}{\mu }\}$ contains all of the solutions to framework (4) that begin in $R_{+}^{2}$. This concludes the proof.

## 3. Equilibrium points and stability analysis

In this part, we get the equilibrium points and existence requirements for the weak (*α*<0) and strong (*α*>0) Allee effects, and we use the Matignon condition [16] to examine their local stability.

In the presence of strong Allee effect (*α*>0), the following biologically feasible equilibrium points are present in system (4): (i) $E_{0}^{S}=(0,0)$, the extinction equilibrium, (ii) $E_{1}^{S}=(\rho ,0)$ $E_{2}^{S}=(\alpha ,0)$, the two axial equilibrium points (iii) If $\alpha <\frac{c}{b}<\rho$ then interior equilibrium point is $E_{3}^{S}=(x_{1}^{*},x_{2}^{*})$ exists, where $x_{1}^{*}=\frac{r}{ab\rho (c+b\beta )}(b\rho -c)(c-\alpha b),x_{2}^{*}=\frac{c}{b}$.

**Theorem 4.** *For any possible equilibrium point with strong Allee effect (α>0)*, *the following stability of the framework (4) is given*:

a) The extinction equilibrium point $E_{0}^{S}=(0,0)$ is remains constant at all times.
b) The axial equilibrium point $E_{1}^{S}=(\rho ,0)$ is stable if $\alpha <\rho <\frac{c}{b}$.
c) The axial equilibrium point $E_{2}^{S}=(\alpha ,0)$ is stable if $\rho <\alpha <\frac{c}{b}$.
d) The interior equilibrium point $E_{3}^{S}=(x_{1}^{*},x_{2}^{*})$ is stable if $\frac{1}{b\beta }-b\beta <c<\frac{(\rho +\alpha )b}{2}$.

**Proof.** This theorem may be proven by defining the community matrix for framework (4), which is shown below

$$J=[\begin{array}{cc} s_{11} & s_{12}\\ s_{21} & s_{22} \end{array}],$$ (5)

where $s_{11}=\frac{r\beta }{{\left(x_{1}+\beta \right)}^{2}}(1-\frac{x_{1}}{\rho })(x_{1}-\alpha )+\frac{rx_{1}}{x_{1}+\beta }(1+\frac{\alpha }{\rho }-\frac{2x_{1}}{\rho })-ax_{2}$, *s*_12_ = −*ax*_1_, $s_{21}=bx_{2},\;s_{22}=bx_{1}-c.$

a) The Jacobian matrix (5) at $E_{0}^{S}=(0,0)$ is defined as

$$J\left(E_{0}^{S}\right)=(\begin{array}{cc} -\frac{\alpha r}{\beta } & 0\\ 0 & -c \end{array}).$$ (6)

The eigen values are $\lambda_{1}=-\frac{\alpha r}{\beta }<0,\lambda_{2}=-c<0$ with $|\arg(\lambda_{i})|=\pi >\frac{\zeta \pi }{2},i=1,2$. Consequently, $E_{0}^{S}$ is locally asymptotically stable according to the Matignon criteria [16].

b) The Jacobian matrix (5) at $E_{1}^{S}=(\rho ,0)$ is defined as

$$J\left(E_{1}^{S}\right)=(\begin{array}{cc} \frac{r\rho }{\rho +\beta }(\frac{\alpha }{\rho }-1) & -a\rho \\ 0 & b\rho -c \end{array}).$$ (7)

The eigen values are $\lambda_{1}=\frac{r\rho }{\rho +\beta }(\frac{\alpha }{\rho }-1)<0$ if *α*<*ρ* and *λ*_2_ = *bρ*−*c*<0 if $\rho <\frac{c}{b}$. Therefore $|\arg(\lambda_{i})|=\pi >\frac{\zeta \pi }{2},i=1,2$ whenever $\alpha <\rho <\frac{c}{b}$. Hence $E_{1}^{S}$ is locally asymptotically stable by the Matignon condition [16].

c) The Jacobian matrix (5) at $E_{2}^{S}=(\rho ,0)$ is defined as

$$J\left(E_{2}^{S}\right)=(\begin{array}{cc} \frac{r\alpha }{\alpha +\beta }(1-\frac{\alpha }{\rho }) & -a\alpha \\ 0 & b\alpha -c \end{array}).$$ (8)

The eigen values are $\lambda_{1}=\frac{r\alpha }{\alpha +\beta }(1-\frac{\alpha }{\rho })<0$ if *ρ*<*α* and *λ*_2_ = *bα*−*c*<0 if $\alpha <\frac{c}{b}$. Therefore $|\arg(\lambda_{i})|=\pi >\frac{\zeta \pi }{2},i=1,2$ whenever $\rho <\alpha <\frac{c}{b}$. Hence $E_{2}^{S}$ is locally asymptotically stable by the Matignon condition [16].

d) The Jacobian matrix at $E_{3}^{S}=(x_{1}^{*},x_{2}^{*})$ is defined in (5). Then $tr(J(E_{3}^{S}))=-[\frac{\beta }{x_{1}+\beta }ax_{2}+\frac{rx_{1}}{x_{1}+\beta }(1+\frac{\alpha }{\rho }-\frac{2x_{1}}{\rho })-ax_{2}]$ and $\det(J(E_{3}^{S}))=abx_{1}x_{2}$. Substituting $x_{1}^{*},x_{2}^{*}$ in above results, we obtain following results. $tr(J(E_{3}^{S}))<0$ if $\frac{1}{b\beta }-b\beta <c<\frac{(\rho +\alpha )b}{2}$ and $\det(J(E_{3}^{S}))>0$ if $\alpha <\frac{c}{b}<\rho$, this is always true for $E_{3}^{S}=(x_{1}^{*},x_{2}^{*})$ exists. Therefore $|\arg(\lambda_{i})|=\pi >\frac{\zeta \pi }{2},i=1,2$ whenever $\frac{1}{b\beta }-b\beta <c<\frac{(\rho +\alpha )b}{2}$. Hence $E_{3}^{S}$ is locally asymptotically stable.

The model system (4) possess the following biologically feasible equilibrium points, for weak Allee effect (*α*<0) (i) $E_{0}^{W}=(0,0)$, the extinction equilibrium, (ii) $E_{1}^{W}=(\rho ,0)$ the axial equilibrium point (iii) If $\frac{c}{b}<\rho$ then interior equilibrium point is $E_{3}^{W}=(x_{1}^{*},x_{2}^{*})$ where $x_{1}^{*}=\frac{r}{ab\rho (c+b\beta )}(b\rho -c)(c-\alpha b),x_{2}^{*}=\frac{c}{b}$.

**Theorem 5.** The stability of the system (4) of each feasible equilibrium points for weak Allee effect (α<0) are as follows:

a) The trivial equilibrium point $E_{0}^{W}=(0,0)$ is always a saddle point.
b) The axial equilibrium point $E_{1}^{W}=(\rho ,0)$ is stable if $\rho <\frac{c}{b}$.
c) The positive equilibrium point $E_{3}^{W}=(x_{1}^{*},x_{2}^{*})$ is stable if $\frac{1}{b\beta }-b\beta <c<\frac{(\rho -\alpha )b}{2}$.

**Proof**.

(a) The community matrix for system (4) may be obtained by replacing (5) with $E_{0}^{W}=(0,0)$, which is represented by
$$J\left(E_{0}^{W}\right)=(\begin{array}{cc} -\frac{\alpha r}{\beta } & 0\\ 0 & -c \end{array}).$$ (9)

The eigen values are $\lambda_{1}=-\frac{\alpha r}{\beta }>0,\lambda_{2}=-c<0$ with $|\arg(\lambda_{1})|=0<\frac{\zeta \pi }{2}$ and $|\arg(\lambda_{2})|=\pi >\frac{\zeta \pi }{2}$ Hence $E_{0}^{W}$ is always a saddle point according to the Matignon criteria [16].

(b) On using $E_{1}^{W}=(\rho ,0)$ to (5), we calculate the community matrix for the system (4)

$$J\left(E_{1}^{W}\right)=(\begin{array}{cc} \frac{r\rho }{\rho +\beta }(\frac{\alpha }{\rho }-1) & -a\rho \\ 0 & b\rho -c \end{array}).$$ (10)

The eigen values are $\lambda_{1}=\frac{r\rho }{\rho +\beta }(\frac{\alpha }{\rho }-1)<0$ and *λ*_2_ = *bρ*−*c*<0 if $\rho <\frac{c}{b}$. Therefore $|\arg(\lambda_{i})|=\pi >\frac{\zeta \pi }{2},i=1,2$ whenever $\rho <\frac{c}{b}$. Hence $E_{1}^{W}$ is locally asymptotically stable by the Matignon condition [16].

(c) The Jacobian matrix at $E_{3}^{W}=(x_{1}^{*},x_{2}^{*})$ is defined in (5). Then $tr(J(E_{3}^{W}))=-[\frac{\beta }{x_{1}+\beta }ax_{2}+\frac{rx_{1}}{x_{1}+\beta }(1-\frac{\alpha }{\rho }-\frac{2x_{1}}{\rho })-ax_{2}]$ and $\det(J(E_{3}^{W}))=abx_{1}x_{2}$. Substituting $x_{1}^{*},x_{2}^{*}$ in above results, we obtain following results. $tr(J(E_{3}^{W}))<0$ if $\frac{1}{b\beta }-b\beta <c<\frac{(\rho -\alpha )b}{2}$ and $\det(J(E_{3}^{W}))>0$ if $\frac{c}{b}<\rho$, this is always true for $E_{3}^{W}=(x_{1}^{*},x_{2}^{*})$ exists. Therefore $|\arg(\lambda_{i})|=\pi >\frac{\zeta \pi }{2},i=1,2$ whenever $\frac{1}{b\beta }-b\beta <c<\frac{(\rho -\alpha )b}{2}$. Hence $E_{3}^{W}$ is locally asymptotically stable.

A Hopf bifurcation will occur in a non-integer order system if its resilience changes and the community matrix computed at the equilibrium point contains a pair of complex conjugate eigenvalues. The criteria initially proposed in [33] are utilised in this instance to determine the presence of a Hopf bifurcation. The order of the non-integer derivative (*ζ*) affects the resilience of the positive equilibrium points for both mild and strong Allee effects, as Theorems 4 and 5 establish. This lets us demonstrate the next theorem, which says that when *ζ* passes over the crucial value ζ*, a Hopf bifurcation has to happen at the positive equilibrium point.

**Theorem 6.** *Let* $\frac{1}{4}{\left(tr(J_{E_{3}^{S}})\right)}^{2}<\det(J_{E_{3}^{S}})$ $\left(or\frac{1}{4}{\left(tr(J_{E_{2}^{W}})\right)}^{2}<\det(J_{E_{2}^{W}})\right)$, *system (4) undergoes a Hopf bifurcation around the interior point $E_{3}^{S}(or\;E_{2}^{W})$ when ζ crosses ζ**.

**Proof.** If $\frac{1}{4}{\left(tr(J_{E_{3}^{S}})\right)}^{2}<\det(J_{E_{3}^{S}})$ eigen values of system (4) at $E_{3}^{S}$ consist of a pair of complex conjugate numbers, both of which have positive real components. We also confirm *ν*_1,2_(ζ*) = 0, where $\nu_{1,2}=\frac{\zeta \pi }{2}-|\arg(\lambda_{i}(m))|$ and ${\frac{\partial \nu_{1,2}}{\partial \zeta }|}_{\zeta =\zeta^{*}}\neq 0$. The equilibrium point $E_{3}^{S}$ experiences a Hopf bifurcation when the parameter *ζ* passes the critical value ζ*, as stated in Theorem 3 in [34].

The same argument applies to the scenario of a mild Allee effect.

### 3.1 Global stability

Under the weak and strong Allee effects, we examine the interior equilibrium $E^{*}=(x_{1}^{*},x_{2}^{*})$’s global asymptotic stability in the following.

**Theorem 7.** *The positive equilibrium point $E^{*}=(x_{1}^{*},x_{2}^{*})$ of framework (4) is globally asymptotically stable*.

**Proof.** Assume the following positive definite Lyapunov function

$$\theta (x_{1},x_{2})=(x_{1}-x_{1}^{*}-x_{1}^{*}\ln(\frac{x_{1}}{x_{1}^{*}}))+\frac{a}{b}(x_{2}-x_{2}^{*}-x_{2}^{*}\ln(\frac{x_{2}}{x_{2}^{*}})).$$ (11)

Clearly *θ*(*x*_1_,*x*_2_) is a positive definite function. Applying the *ζ*^*th*^ order Caputo non-integer derivative on both sides of the Eq (11) we obtain,

$${\;}^{C}D_{t}^{\zeta }\theta (x_{1},x_{2}){=}^{C}D_{t}^{\zeta }(x_{1}-x_{1}^{*}-x_{1}^{*}\ln(\frac{x_{1}}{x_{1}^{*}}))+\frac{a}{b}{\;}^{C}D_{t}^{\zeta }(x_{2}-x_{2}^{*}-x_{2}^{*}\ln(\frac{x_{2}}{x_{2}^{*}})).$$ (12)

Lemma 4 in Reference [31] provides us with a relation,

$${\;}^{C}D_{t}^{\zeta }\theta (x_{1},x_{2})\leq -(\frac{r}{\rho (x_{1}+\beta )}\left(x_{1}-x_{1}^{*}\right)\left(x_{1}-\rho \right)\left(x_{1}-\alpha \right)).$$ (13)

Now we construct the domain $D=\{(x_{1},x_{2}):(x_{1}-x_{1}^{*})(x_{1}-\alpha )<0\}$. Then, clearly ${\;}^{C}D_{t}^{\zeta }\theta (x_{1},x_{2})\leq 0$ for all (*x*_1_,*x*_2_) belongs to the set *D* as *x*_1_,*x*_2_>0 and ${\;}^{C}D_{t}^{\zeta }\theta (x_{1},x_{2})=0$ specifies that $x_{1}=x_{1}^{*}$. Following that, $x_{2}=x_{2}^{*}$ is the equilibrium condition. Therefore, the singleton set {E*} is the only invariant set for which ${\;}^{C}D_{t}^{\zeta }\theta (x_{1},x_{2})=0$. Lemma 4.6 from Reference [35] implies that the equilibrium point $E^{*}=(x_{1}^{*},x_{2}^{*})$ is globally stable in region $D=\{(x_{1},x_{2}):(x_{1}-x_{1}^{*})(x_{1}-\alpha )<0\}$. For weak Allee effect the domain $D=\{(x_{1},x_{2}):x_{1}<x_{1}^{*}\}$.

## 4. Numerical simulations

**Fig 1:**
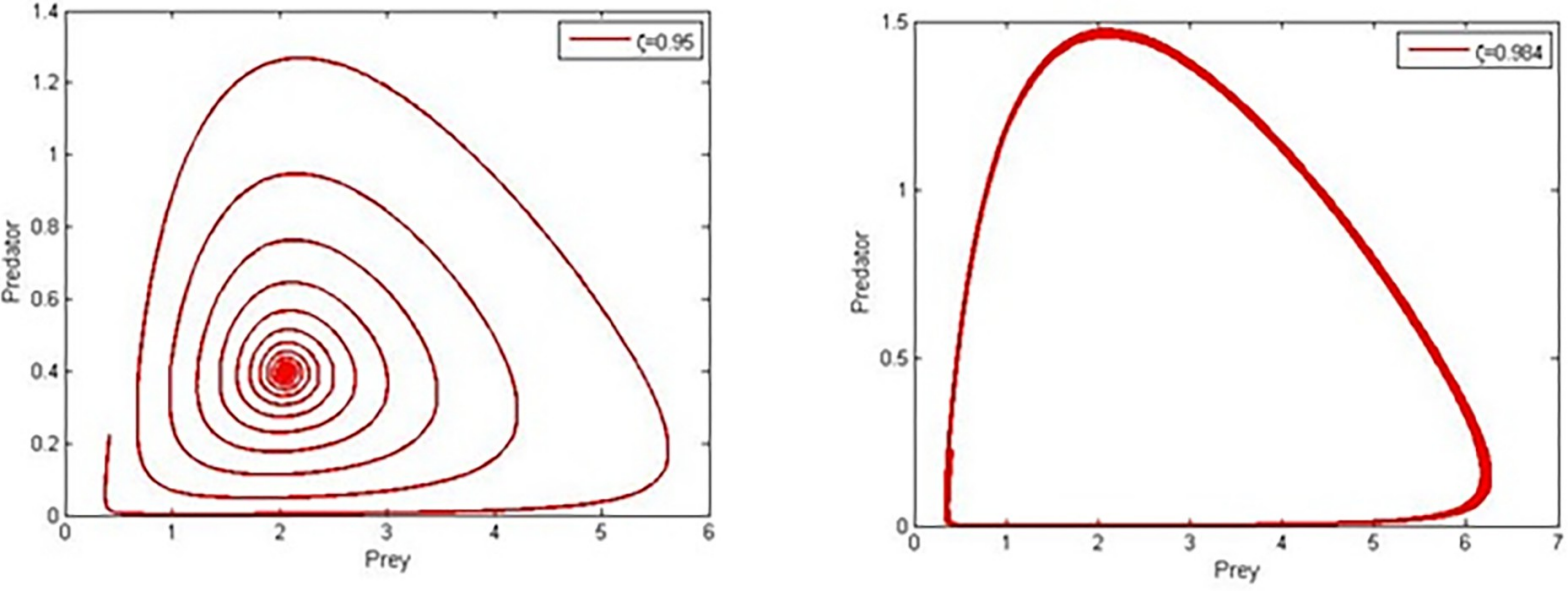
Phase portraits of system (4) for different fractional order values.

**Fig 2:**
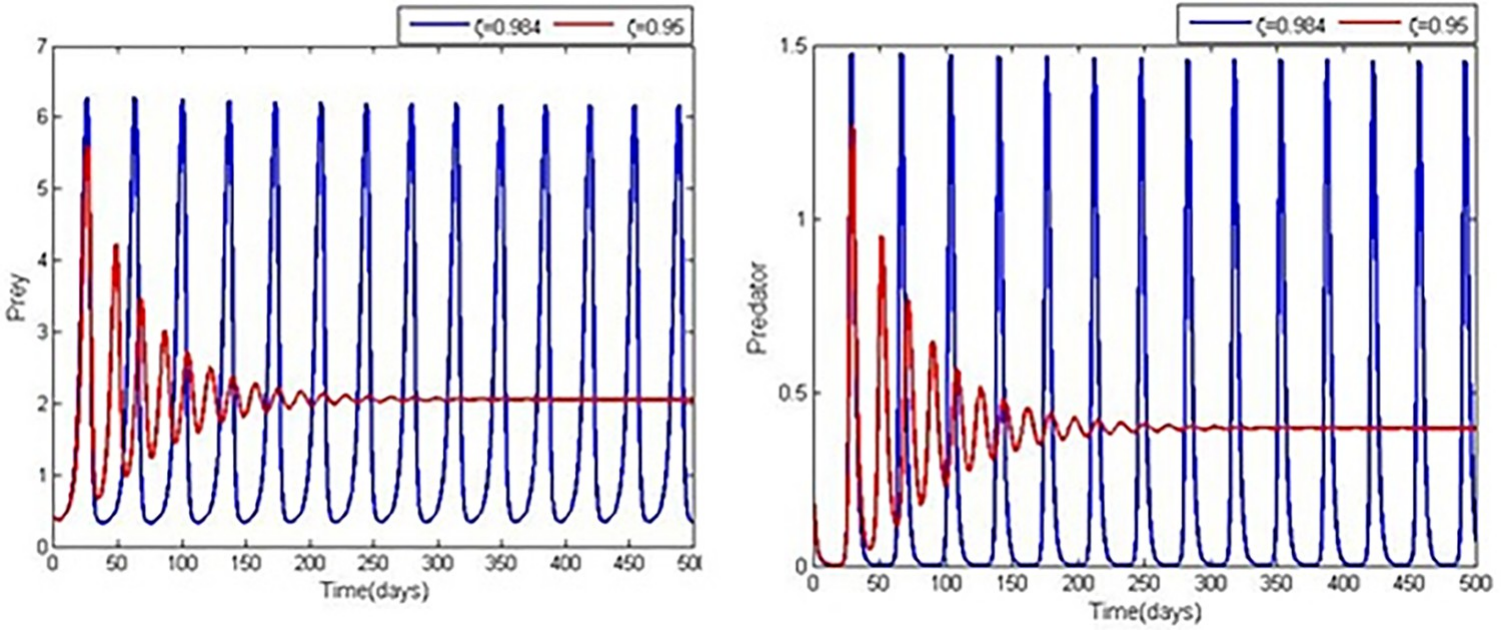
Time series of system (4) for different fractional order values.

We analyse system (4) using the below hypothetical parameter values r=0.528,ρ=8,α=0.289,β=0.7290,a=0.627,b=0.276,c=0.567, Adams-Bashforth-Moulton algorithm [36], and run simulations to explore the impact of the order of the non-integer derivative (*ζ*). In light of the parameter values mentioned earlier and the results of Theorem 6, we can establish that the positive equilibrium point is asymptotically stable while *ζ*is less than ζ*and destabilising when *ζ* is more than ζ* by setting the crucial value ζ* to be 0.98. The asymptotic stability of the positive equilibrium point $E_{3}^{s}=(2.0543,0.3970)$ is observed to be present when *ζ* is smaller than ζ*. However, when *ζ* exceeds ζ*, the stability of the positive equilibrium point $E_{3}^{s}$ deteriorates, resulting in the convergence of all numerical solutions to a limit-cycle through a Hopf bifurcation. The Hopf bifurcation may be noticed based on the phase-portraits and trajectories shown in both Figs 1 and 2 given *ζ* = 0.95<ζ* and for*ζ* = 0.984>ζ*, respectively.

We next use the following hypothetical parameter values to analyse numerically how the Allee criterion *α*>0 affects the behaviour of system (4), *r* = 0.067, *ρ* = 7.65, α=0.31,β=0.02,a=0.289,b=0.482,c=0.467.

**Fig 3:**
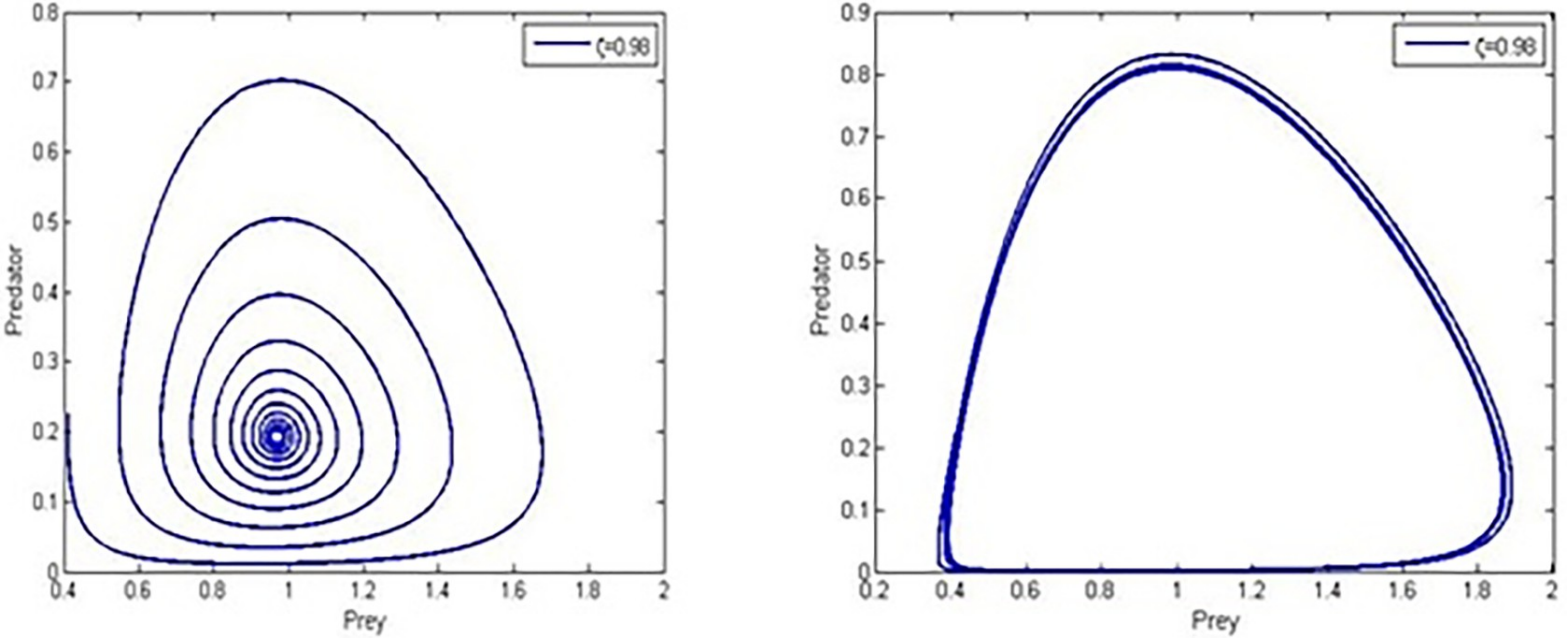
Phase portraits of system (4) for strong Allee effect *α* = 0.02,0.31.

If we take a look at the parameter values mentioned before, we can determine a critical value α* = 0.19 that makes positive equilibrium point asymptotically stable if *α*<α* and not stable if *α*>α*. All numerical solutions converge to a limit-cycle through a Hopf bifurcation once the interior point $E_{3}^{s}$ loses its stability, $E_{3}^{s}=(0.9689,0.1349)$ which is asymptotically stable for *α*<α* and unstable for *α*>α*. The phase-portraits for *α* = 0.02<α* and *α* = 0.31>α* in Fig 3 also demonstrates the Hopf bifurcation.

**Fig 4:**
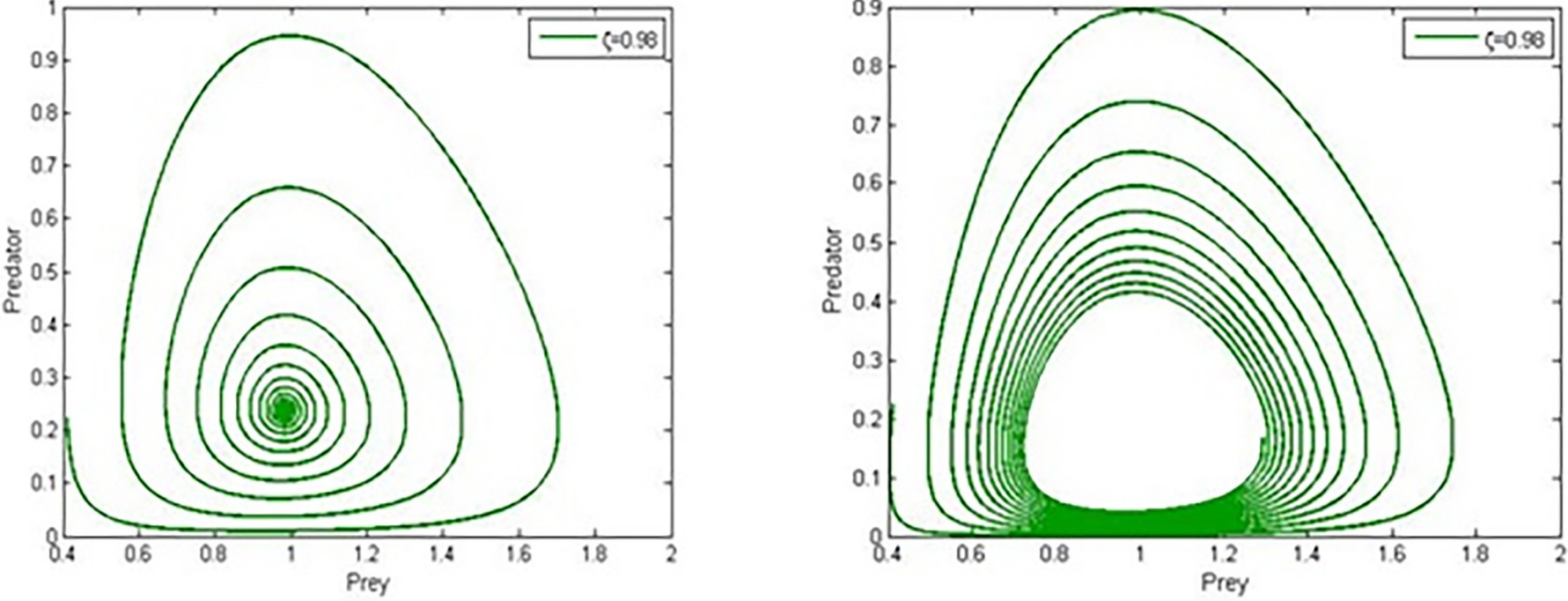
Phase portraits of system (4) for weak Allee effect *α* = −0.5,−0.01.

We achieved a critical value α* = −0.3868 and found the Hopf bifurcation result (shown in Fig 4). provided the influence of Allee is weak (*α*<0). In this case the parameter values are r=0.077,ρ=9.56,β=0.5,a=0.289,b=0.682,c=0.67 and corresponding to this equilibrium point is $E_{2}^{w}=(0.9824,0.1600)$.

## 5. Conclusions

This work presented and analysed the dynamics of a fractional-order predator-prey system with Holling type-I functional response and double Allee effect in the prey population. The combined impact accelerates the probability of extinction and population decline, and more theoretical research is required to support the coexistence of these varied ecosystems of vulnerable populations [37]. Therefore, when managing exploited or threatened populations, the multiple Allee effect is more important than the single Allee effect from an ecological perspective. By using Adams-Bashforth-Moulton algorithm and parametric values r=0.077,ρ=9.56,β=0.5,a=0.289,b=0.682,c=0.67, we studied different scenarios and phase-portraits for the prey and predator, Fig 1. *ζ* = 0.95<ζ*, and *ζ* = 0.984>ζ* for Predator, Fig 2. *ζ* = 0.984, 0.95 for prey and *ζ* = 0.984, 0.95 for prey and predator, Fig 3. *ζ* = 0.98, *α* = 0.02,0.31 predator and prey, and Fig 4. *ζ* = 0.98, *α* = 0.02,0.31 for prey and predator.

Initially, it has been shown that the solution exists, is unique, non-negativity, and is bounded. The local and global stability features of each potential non-negative equilibrium point were then determined. The extinction of prey and predator points continues to be stable in strong Allee effect situations, but unstable in weak Allee influence scenarios. When $\alpha <\rho <\frac{c}{b}$, the predator extinction threshold remains stable in the strong Allee effect scenario; however, when $\rho <\frac{c}{b}$, it remains stable under the weak Allee effect case. On top of that, we established that there exists a Hopf bifurcation governed by the order of the non-integer derivative (*ζ*) surrounding the interior point and we analytically determined the critical ζ* of this bifurcation. We confirm that the Hopf bifurcation occurs in our numerical simulations. In addition, computer simulations have shown that Hopf bifurcation occurs in both strong and weak Allee effects. In these cases, the double Allee effect-affected predator population may be able to reside in our system (4).

According to the results shown above, the dynamical behaviour of this system can be controlled and predicted by paying close attention to the sensitivity of the extinction, coexistence, and oscillation phenomena in the two populations that make up the biological model that incorporates the double Allee effect.
